# Comparison of Various Solvent Extracts and Major Bioactive Components from *Portulaca oleracea* for Antioxidant, Anti-Tyrosinase, and Anti-α-Glucosidase Activities

**DOI:** 10.3390/antiox11020398

**Published:** 2022-02-16

**Authors:** Wei-Cheng Chen, Shih-Wei Wang, Cai-Wei Li, Hsiang-Ru Lin, Chang-Syun Yang, Yi-Cheng Chu, Tzong-Huei Lee, Jih-Jung Chen

**Affiliations:** 1 Department of Medicine, MacKay Medical College, New Taipei City 252, Taiwan; wchena7648@gmail.com (W.-C.C.); shihwei@mmc.edu.tw (S.-W.W.); 2 Division of Sports Medicine & Surgery, Department of Orthopedic Surgery, MacKay Memorial Hospital, Taipei 104, Taiwan; 3 Institute of Biomedical Sciences, MacKay Medical College, New Taipei City 252, Taiwan; 4 Graduate Institute of Natural Products, Kaohsiung Medical University, Kaohsiung 807, Taiwan; 5 Institute of Traditional Medicine, National Yang Ming Chiao Tung University, Taipei 112, Taiwan; leecw1219.y@nycu.edu.tw (C.-W.L.); chuyc.md07@nycu.edu.tw (Y.-C.C.); 6 Department of Chemistry, College of Science, National Kaohsiung Normal University, Kaohsiung 824, Taiwan; t3136@nknu.edu.tw; 7 Department of Pharmacy, School of Pharmaceutical Sciences, National Yang Ming Chiao Tung University, Taipei 112, Taiwan; tim0619@nycu.edu.tw; 8 Institute of Fisheries Science, National Taiwan University, Taipei 106, Taiwan; 9 Department of Medical Research, China Medical University Hospital, China Medical University, Taichung 404, Taiwan

**Keywords:** *Portulaca oleracea*, various solvent extracts, major bioactive components, antioxidant activity, anti-α-glucosidase activity, anti-tyrosinase activity

## Abstract

*Portulaca oleracea* is a well-known species for traditional medicine and food homology in Taiwan. In traditional medicine, *P. oleracea* is also used to treat gastrointestinal disorders, liver inflammation, fever, severe inflammation, and headaches. We investigated antioxidant, anti-tyrosinase, and anti-α-glucosidase activities of various solvent extracts and major bioactive components from *P. oleracea*. Ethanol and acetone extracts showed potent DPPH, ABTS, and hydroxyl radical scavenging activities. Chloroform and *n*-hexane extracts displayed significant superoxide radical scavenging activity. Furthermore, ethyl acetate and acetone extracts of *P. oleracea* showed potent anti-tyrosinase and anti-α-glucosidase activities. Examined and compared to the various solvent extracts for their chemical compositions using HPLC analysis, we isolated seven major compounds and analyzed their antioxidant, anti-tyrosinase, and anti-α-glucosidase activities. Seven active compounds of *P. oleracea*, especially quercetin, rosmarinic acid, and kaempferol, exhibited obvious antioxidant, anti-tyrosinase, and anti-α-glucosidase activities. The molecular docking model and the hydrophilic interactive mode of tyrosinase and α-glucosidase revealed that active compounds might have a higher antagonistic effect than commonly inhibitors. Our result shows that the active solvent extracts and their components of *P. oleracea* have the potential as natural antioxidants, tyrosinase and α-glucosidase inhibitors. Our results suggest that the active solvent extracts of *P. oleracea* and their components have potential as natural antioxidants, tyrosinase and α-glucosidase inhibitors.

## 1. Introduction

Free radicals are substances produced after oxygen metabolism in the body, and are molecules or molecular fragments whose atoms contain unpaired electrons or contain other oxygen species, including molecular orbitals or simple reactive oxygen species, which are very active and can react strongly with any substance [1]. An imbalance between the production and quenching of free radicals from oxygen species leads to oxidative stress. Furthermore, these reactive oxygen species (ROS) play an important role in many chronic diseases including mitochondrial diseases [2]. The adverse effects of oxidative stress on human health have become a serious issue.

Numerous studies have shown an inverse relationship between the dietary intake of antioxidant-rich foods and incidence of human disease [3]. However, synthetic antioxidants, such as butylated hydroxyanisole (BHA) and butylated hydroxytoluene (BHT), have been widely used as antioxidants in the food industry and may cause liver damage and carcinogenesis [4,5]. This reason has sparked public interest in natural antioxidants as an alternative.

Disturbed carbohydrate metabolism contributes to harmful health problems such as obesity, oral disease and diabetes worldwide. Recent studies have shown that oxidative damage plays a major role in the occurrence of many degenerative and chronic diseases, and oxidative damage to pancreatic islet tissue can lead to diabetes [6]. Furthermore, type 2 diabetes mellitus (T2DM) is a severe chronic metabolic disease that accounts for more than 90% of all diabetic patients [7], and diet plays an important role in the development of T2DM [8]. It has been reported that regulating and inhibiting the activities of major digestive enzymes, especially pancreatic α-amylase and α-glucosidase, can reduce the rate of carbohydrate absorption in the gastrointestinal tract, control or even prevent T2DM, and relieve postprandial hyperglycemia [9].

When it comes to enzymes related to oxidation, tyrosinase is also a common oxidase. Tyrosinase is a copper-containing enzyme mainly involved in the biosynthesis of melanin. It catalyzes melanin biosynthesis in human skin, and epidermal hyperpigmentation may lead to various skin diseases such as age spots and melasma [10,11]. In addition, tyrosinase also plays an important role in the food industry. Tyrosinase catalyzes enzymatic browning in plants, which may cause adverse changes in the appearance, flavor, texture, and nutritional value of plant-derived foods and beverages [12]. Various synthetic and natural compounds, such as ascorbic acid and 4-hexylresorcinol, have been developed to inhibit enzymatic browning. However, their use has some limitations due to their temporary action and the requirement for combined action of reducing agents [13,14,15]. Recently, safe and effective tyrosinase inhibitors have become important for their potential applications in improving food quality and preventing pigmentation disorders and other melanin-related health problems in humans [16]. Natural tyrosinase inhibitors are generally considered to have no deleterious side effects and can be produced at reasonable low cost, especially when abundant sources are identified.

*Portulaca oleracea* L. (PO) is an annual herb with reddish stems and alternate leaves from the Portulacaceae family. It is distributed in many parts of the world, especially in tropical and subtropical regions [17]. *P. oleracea* has been used as a traditional remedy for a variety of ailments, including gastrointestinal disorders, respiratory disorders, liver inflammation, kidney and bladder ulcers, fever, insomnia, severe inflammation, headaches, etc. In our studies on the antioxidant and anti-inflammatory activities of plants in Taiwan, many species have been screened for these effects, and *P. oleracea* was found to be an active species. This report describes the evaluation of the antioxidant, anti-tyrosinase, and anti-α-glucosidase activities of various solvent extracts from *P. oleracea* and their main bioactive components.

## 2. Materials and Methods

### 2.1. Chemicals and Antibodies

Nicotinamide adenine dinucleotide (NADH), trichloroacetic acid (TCA), and butyl hydroxytoluene (BHT) were obtained from Acros Organics (Geel, Belgium). *p*-Nitro-phenyl-α-*D*-glucopyranoside (*p*-NPG) and ferric chloride (FeCl_3_) were purchased from Alfa Aesar (Lancashire, UK). Folin–Ciocalteu’s reagent, ethylenediaminetetraacetic acid (EDTA), 2,2′-azino-bis(3-ethylbenzothiazoline-6-sulfonic acid) (ABTS), tyrosinase, α-glucosidase, hydrogen peroxide solution, and ascorbic acid were supplied by Sigma-Aldrich (St. Louis, MO, USA). Potassium peroxodisulfate, sodium carbonate, disodium hydrogen phosphate, and potassium dihydrogen phosphate were obtained from the SHOWA Chemical Co. Ltd. (Chuo-ku, Japan). Phenazine methosulfate (PMS), 2,2-diphenyl-1-(2,4,6-trinitrophenyl)hydrazyl (DPPH), nitro blue tetrazolium (NBT), phloroglucinol, deoxyribose, and 2-thiobarbituric acid (TBA) were purchased from Tokyo Chemical Industry Co., Ltd. (Tokyo, Japan).

### 2.2. Preparation of P. oleracea Extract

The whole plant of *P. oleracea* was collected from Xichang Dist., Taipei City, Taiwan, in September 2020 and identified by Prof. J.-J. Chen. A voucher specimen was deposited in the Department of Pharmacy, National Yang Ming Chiao Tung University, Taipei, Taiwan. Samples were collected, air-dried, and ground to powder (0.5 to 2 mm). Preparation of *P. oleracea* extract was performed as described previously [18].

### 2.3. Preparation of Active Components

The dried whole plant (250 g) of *P. oleracea* was pulverized and extracted three times with MeOH (0.5 L each) for 3 days. The MeOH extract was concentrated under reduced pressure at 37 °C, and the residue (95 g) was partitioned between EtOAc and H_2_O (1:1) to provide the EtOAc-soluble fraction (fraction A, 32.3 g). Fraction A (32.3 g) was chromatographed on silica gel (70–230 mesh, 1450 g), eluting with *n*-hexane, gradually increasing the polarity with EtOAc to give 10 fractions: A1–A10. Fraction A3 (4.06 g) was further isolated by silica gel chromatography column (CC) and purified by preparative TLC (silica gel; *n*-hexane/acetone, 1:1) to afford kaempferol (4.40 mg), *p*-coumaric acid (6.22 mg), and caffeic acid (3.85 mg). Part (210 mg) of fraction A6 was further purified by preparative TLC (silica gel; dichloromethane/EtOAc, 6:1) to afford quercetin (14.40 mg). Part (195 mg) of fraction A8 was further isolated by preparative TLC (silica gel; dichloromethane/methanol, 3:1) to afford rosmarinic acid (4.52 mg). Part (180 mg) of fraction A9 was further purified by preparative TLC (silica gel; dichloromethane/methanol, 2:1) to afford chlorogenic acid (5.52 mg).

### 2.4. Reverse-Phase HPLC

Reversed-phase separations were performed using a LiChrospher^®^ 100 RP-18 Endcapped (5 μm; column of dimensions 4.6 × 250 mm) purchased from Merck KGaA, Darmstadt, Germany. HPLC-PDA chromatographic fingerprints were obtained with an Agilent 1260 Infinity II HPLC instrument equipped with a 1260 Infinity II quaternary pump, a 1260 Infinity II degasser, a 1260 Infinity II vialsampler, a 1260 Infinity II column thermostat, a 1260 Infinity II diode array detector HS, and a PC with the Agilent ChemStation software. All of them were purchased from Agilent Technologies (Waldbronn, Germany). Gradient separation using 0.2% acetic acid in water (*v*/*v*) (solvent A) and acetonitrile (solvent B) as mobile phase was as follows: 0–15 min, linear gradient from 95 to 88% A; 15–35 min, 88% A with isocratic elution; 35–55 min, 75% A with isocratic elution; 55–65 min, 60% A with isocratic elution; 65–90 min, 40% A with isocratic elution; 90–95 min, linear gradient from 40 to 0% A; 95–100 min, back to initial condition at 95% A; and 100–105 min, at 95% A. The flow rate was 1.0 mL/min, and the injection volume was 500 μL. Peaks were detected at 280 nm. Different compounds were identified by retention time. To guarantee peak purity, DAD acquisition from 200–650 nm was conducted to register UV-spectra. For the quantitative analysis of seven compounds in the extracts, aliquots of samples were dispersed in 10 mL of a methanol solution by sonication for 5 min. Following this, the samples were centrifuged for 15 min at 3500 rpm, and the supernatant extracts were filtered through 0.45 μm PTFE syringe filters (Zhejiang Sorfa Medical Plastic Co., Ningbo, China). Quantification of seven components from *Portulaca oleracea* in each solvent extract was described as above.

### 2.5. Determination of Total Phenolic Content

The total phenolic content (TPC) of various solvent extracts was measured as described with Folin–Ciocalteu’s method with a slight modification [18].

### 2.6. Determination of Total Flavonoid Content

The total flavonoid content (TFC) of different solvent extracts was determined with slight modification of the aluminum chloride colorimetric method described by Chang et al. [19]. In brief, the extract sample was dissolved with ethanol to 10 mg/mL. The calibration curve was prepared by diluting quercetin in methanol (0–100 mg/mL). The dissolved extract or quercetin (2.0 mL) was mixed with 0.1 mL of 10% (*w*/*v*) aluminum chloride solution and 0.1 mL of 0.1 mM potassium acetate solution. The mixture was kept at room temperature (25 °C) for 30 min. Then the maximum absorbance of the mixture was measured at 415 nm using a UV–VIS spectrophotometer. The TFC was expressed as milligram quercetin equivalent per gram of dry extract (mg QCE/g dry extract).

### 2.7. DPPH Radical Scavenging Activity

The DPPH radical scavenging assay was determined by the reference method [18].

### 2.8. ABTS Cation Radical Scavenging Activity

The ABTS cation radical scavenging assay was carried out using the reference method [18].

### 2.9. Superoxide Anion Radical Scavenging Activity

Superoxide anion radical (O_2_^•−^) scavenging activity was described earlier with slight changes [18]. Briefly, the superoxide radical was generated in 16 mM Tris-HCl buffer (pH 8.0) containing 50 μL of NBT (300 μM), 50 μL of PMS (120 μM), and 50 μL of different concentrations of test sample. The reaction was initiated by adding 50 μL of NADH (468 μM) solution to the mixture. After incubating at room temperature for 5 min, the activity of samples was determined by calculating the decrease in absorbance measured at 560 nm by the following equation,

Superoxide anion radical scavenging activity (%) = (A_0_ − A_1_)/A_0_ × 100, where A_1_ is the absorbance of the test sample and A_0_ is the absorbance of the control.

IC_50_ values for the tested activities were determined by linear regression of the percentage of remaining superoxide anion radicals against sample concentration.

### 2.10. Hydroxyl Radical Scavenging Activity

The samples were evaluated for hydroxyl radical scavenging activity based on the previously described method [18].

### 2.11. Anti-Tyrosinase Activity Assay

The substrate was premixed with the sample at various concentrations in potassium phosphate buffer (50 mM, pH 6.5). After incubating at room temperature for 10 min, 20 μL of mushroom tyrosinase (1000 units/mL) was added, and the mixtures were incubated for another 30 min. Absorbance was measured at 490 nm and the percentage of anti-tyrosinase activity of sample was calculated from,

Inhibition (%) = (A_0_ − A_1_)/A_0_ × 100, where A_1_ is the absorbance of the test sample and A_0_ is the absorbance of the control. Arbutin was used as positive control.

### 2.12. Anti-α-Glucosidase Activity Assay

α-Glucosidase inhibitory activity was established in accordance with the described method [18] with slight modifications. In brief, 100 μL of different concentrations of the sample, 380 μL of *p*-nitro-phenyl-α-D-glucopyranoside (*p*-NPG) (0.53 mM), and 20 μL of α-Glucosidase solution (1 units/mL) were mixed in 0.1 M potassium phosphate buffer (pH 6.8). After incubating at 37 °C for 20 min, 500 μL of sodium carbonate (0.1 M) was added in order to quench the reaction. The anti-α-glucosidase activity of the sample was determined by calculating the decrease in absorbance measured at 405 nm by the following equation,

Inhibition (%) = (A_0_ − A_1_)/A_0_ × 100, where A_1_ is the absorbance of the test sample and A_0_ is the absorbance of the control. Acarbose was used as positive control.

### 2.13. Molecular Modeling Docking Study

All calculations were performed by Discovery Studio 2019 (San Diego, CA, USA) software. Primarily, the structure of the docked compound is energy minimized until the default derivative convergence criterion of 0.01 kcal/mol is met. The crystal structure (PDB: 3A4A or 2Y9X) is retrieved from the Protein Databank and hydrogen atoms are added to prepare the docked receptor. This protein structure is subsequently used in the CDocker program to dock the compound into the active site. Ten different docking poses are calculated and ranked by using the PLP score scoring function. The top-ranked docking solution is visually analyzed to determine the binding mode of the docked compound.

### 2.14. Statistical Analysis

All data are expressed as mean ± SD. Statistical analysis was carried out using the Mann–Whitney *U* test. A probability of 0.05 or less was considered statistically significant. All the experiments were performed at least 3 times.

## 3. Results

### 3.1. Determination of TPC, TFC, and Yields in Each Solvent Extract

We investigated the total phenol contents (TPC), total flavonoid contents (TFC) and yields in different solvent extracts of *P. oleracea*. Table 1 shows TPC, TFC and extraction yields of *n*-hexane, chloroform, dichloromethane, ethyl acetate, acetone, methanol, and ethanol extracts from *P. oleracea*. The yields of different solvent extracts from *P. oleracea* ranged from 19.2 ± 1.25% (chloroform extract) to 38.2 ± 1.43% (methanol extract). We found TPC in all the different solvent extracts, with the ethanol extract having the highest amount of TPC (219.27 ± 4.13 mg/g), followed by acetone (34.61 ± 4.87 mg/g), ethyl acetate (30.91 ± 3.09 mg/g), dichloromethane (30.12 ± 2.79 mg/g), chloroform (28.99 ± 1.30 mg/g), methanol (27.80 ± 2.86 mg/g) and *n*-hexane (19.67 ± 3.33 mg/g).

On the other hand, differences were found in TFC among different solvent extracts, of which ethanol extract contained the highest amount of TFC (437.38 ± 13.14 mg/g) followed by ethyl acetate (115.49 ± 8.85 mg/g), dichloromethane (107.75 ± 7.28 mg/g), acetone (89.65 ± 8.53 mg/g), chloroform (66.60 ± 5.81 mg/g), *n*-hexane (36.41 ± 5.81 mg/g), and methanol (27.21 ± 0.74 mg/g). The TPC of the ethanolic extract was about 7 to 11 times higher than that of other solvent extracts, and the TFC was about 4 to 16 times higher, indicating that the suitable relative polarity of the solvent for extracting phenolics and flavonoids from *P. oleracea* is about 0.654.

The comparative evaluation of the total phenolic content (TPC) and total flavonoid content (TFC) of various solvent extracts (*n*-hexane, chloroform, dichloromethane, ethyl acetate, acetone, ethanol, and methanol) from the whole plant of *P. oleracea* is first conducted in our study. This can provide a guide for the selection of appropriate solvents in TPC and TFC extraction applications.

### 3.2. DPPH Free-Radical Scavenging Activities of Different Solvent Extracts

DPPH is relatively stable in aqueous or ethanol solution. Antioxidants could interact with DPPH radical and transfer an electron or hydrogen atom to DPPH radical so as to neutralize free radicals [20]. The DPPH free-radical scavenging activity of each extract is shown in Table 2, and the ethanolic extract (IC_50_ = 41.33 ± 2.89 μg/mL) exhibited DPPH free-radical scavenging activity, which was comparable to BHT (IC_50_ = 39.88 ± 1.30 μg/mL). In addition, acetone (IC_50_ = 49.79 ± 3.34 μg/mL), methanol (IC_50_ = 55.36 ± 2.08 μg/mL) and ethyl acetate (IC_50_ = 64.24 ± 3.03 μg/mL) extracts also exhibited DPPH scavenging activity.

### 3.3. ABTS Free-Radical Scavenging Activities of Different Solvent Extracts

As shown in Table 2, ethyl acetate (IC_50_ = 45.40 ± 6.06 μg/mL) and acetone (IC_50_ = 45.40 ± 6.06 μg/mL) extracts exhibited stronger ABTS radical scavenging activity, followed by ethanol (IC_50_ = 64.69 ± 3.97 μg/mL), methanol (IC_50_ = 69.85 ± 6.76 μg/mL), dichloromethane (IC_50_ = 79.03 ± 6.52 μg/mL), chloroform (IC_50_ = 97.91 ± 7.36 μg/mL) and *n*-hexane (IC_50_ = 296.79 ± 10.16 μg/mL).

### 3.4. Superoxide Radical Scavenging Activities of Different Solvent Extracts

The extracts of chloroform (IC_50_ = 6.80 ± 1.77 μg/mL) and *n*-hexane (IC_50_ = 14.36 ± 2.17 μg/mL) exhibited strong superoxide radical scavenging activities (Table 2). However, the results showed that other extracts had no significant effect on superoxide radical scavenging activity (IC_50_ > 400 μg/mL).

### 3.5. Hydroxyl Radical Scavenging Activities of Different Solvent Extracts

The ethanol extract showed the strongest activity for scavenging hydroxyl radical (IC_50_ = 23.23 ± 0.28 μg/mL), followed by methanol (IC_50_ = 3n8.31 ± 0.54 μg/mL), ethyl acetate (IC_50_ = 68.60 ± 5.76 μg/mL), acetone (IC_50_ = 85.92 ± 5.99 μg/mL), dichloromethane (IC_50_ = 115.66 ± 17.68 μg/mL), chloroform (IC_50_ = 115.95 ± 7.01 μg/mL) and *n*-hexane (IC_50_ = 132.94 ± 12.85 μg/mL).

Comparative evaluation of antioxidant assays (DPPH, ABTS, superoxide, and hydroxyl radical scavenging) of various solvent extracts (*n*-hexane, chloroform, dichloromethane, ethyl acetate, acetone, ethanol, and methanol) from the whole plant of *P. oleracea* is first proposed in our study. This can provide an indication for the selection of appropriate solvents in antioxidant extraction applications.

### 3.6. Anti-Tyrosinase Activities of Different Solvent Extracts

Tyrosinase inhibitors are considered important for their potential applications in improving food quality and preventing pigmentation disorders and other melanin-related health problems in humans. As shown in Table 3, among all extracts, ethyl acetate (IC_50_ = 396.20 ± 24.73 μg/mL) and acetone (IC_50_ = 412.22 ± 9.52 μg/mL) extracts of *P. oleracea* showed more potent anti-tyrosinase activity, followed by ethanol (IC_50_ = 497.24 ± 32.99 μg/mL), dichloromethane (IC_50_ = 545.29 ± 29.29 μg/mL), methanol (IC_50_ = 571.35 ± 25.73 μg/mL), *n*-hexane (IC_50_ = 621.43 ± 41.33 μg/mL), and chloroform (IC_50_ = 659.87 ± 24.89 μg/mL).

### 3.7. Anti-α-Glucosidase Activities of Different Solvent Extracts

Natural products with anti-α-glucosidase activity have received extensive attention for their potential use in the treatment of diabetes. As shown in Table 3, the acetone extract of *P. oleracea* had the highest anti-α-glucosidase activity (IC_50_ = 328.24 ± 52.67 μg/mL), followed by dichloromethane (IC_50_ = 416.34 ± 13.58 μg/mL), *n*-hexane alkane (IC_50_ = 418.31 ± 11.95 μg/mL), ethanol (IC_50_ = 440.31 ± 5.94 μg/mL), ethyl acetate (IC_50_ = 472.92 ± 2.45 μg/mL), chloroform (IC_50_ = 527.06 ± 25.64 μg/mL), and methanol (IC_50_ > 800 μg/mL). The acetone extract has comparable anti-α-glucosidase activity compared to the positive control acarbose (IC_50_ = 319.52 ± 24.46 μg/mL).

The comparative assessment of anti-tyrosinase and anti-α-glucosidase assays of various solvent extracts (*n*-hexane, chloroform, dichloromethane, ethyl acetate, acetone, ethanol, and methanol) from the whole plant of *P. oleracea* is first conducted in our study. This can provide an indication for the selection of appropriate solvents in natural tyrosinase and α-glucosidase inhibitors extraction applications.

### 3.8. Quantitation of Active Components in Different Solvent Extracts

The HPLC methods using reverse-phase column for the quantification of seven components isolated from *P. oleracea* were verified regarding linearity, LOD, and LOQ. The linearity was validated by the data from six different concentrations (1.0, 5.0, 10.0, 25.0, 50.0, and 100.0 μg/mL) of the standard solutions. The linear regression parameters of calibration curves, correlation coefficient, LOD, and LOQ were shown in Table S1. Six concentrations of each standard were analyzed in triplicate to generate respective calibration curve. The linearity (*R*^2^ > 0.9993) between Y (the peak area of the analytes with external standard) and X (concentration of the standards) was achieved in the tested stage.

**Figure 1:**
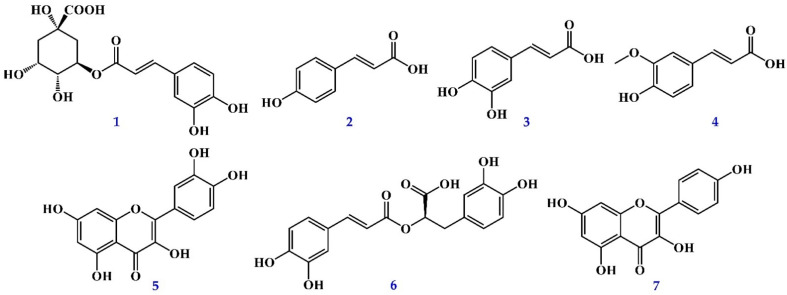
The chemical structures of seven compounds from *P. oleracea*.

Figures S1–S8 displayed the quantification of active components in different solvent extracts from *P. oleracea* by reverse-phase HPLC analyses. The contents of seven compounds in each solvent extract were shown in Table 4. Total quantities of seven compounds in each extract ranged from a maximum of 50.90 ± 1.55 mg/kg (EtOH extract) to a minimum of 10.85 ± 0.66 mg/kg (EtOAc extract) in succeeding order of ethanol > chloroform > methanol > *n*-hexane > dichloromethane > acetone > ethyl acetate. The EtOH extract exhibited more of the seven active components than the other extracts. Among the seven active compounds in the organic solvent extract, kaempferol was the most abundant, followed by quercetin, *trans*-ferulic acid, chlorogenic acid, *p*-coumaric acid, caffeic acid, and rosmarinic acid (Figure 1).

### 3.9. Antioxidant Activities of Isolated Components

Compounds isolated from *P. oleracea* were measured for their antioxidant activities, including DPPH, ABTS, superoxide, and hydroxyl radical scavenging assays. The results are shown in Table 5. Except for *p*-coumaric acid, most of the compounds have much higher DPPH free-radical scavenging activity than the positive control BHT (IC_50_ = 31.04 ± 2.12 μg/mL). Furthermore, all seven compounds showed high ABTS radical scavenging activity, especially kaempferol (IC_50_ = 8.75 ± 0.90 μg/mL) and quercetin (IC_50_ = 11.46 ± 1.82 μg/mL). In the superoxide radical scavenging assay, quercetin (IC_50_ = 44.08 ± 3.07 μg/mL) showed the strongest activity, followed by caffeic acid (IC_50_ = 57.34 ± 7.44 μg/mL) and rosmarinic acid (IC_50_ = 61.62 ± 6.43 μg/mL). In addition, rosmarinic acid (IC_50_ = 6.56 ± 4.77 μg/mL), quercetin (IC_50_ = 9.75 ± 4.39 μg/mL) and kaempferol (IC_50_ = 11.46 ± 5.60 μg/mL) showed strong hydroxyl radical scavenging activity.

In conclusion, all seven compounds isolated from *P. oleracea* exhibited good antioxidant activity against DPPH, ABTS, superoxide, and hydroxyl radical scavenging. The comparative evaluation of antioxidant assays of active compounds from the whole plant of *P. oleracea* is first conducted in our study.

### 3.10. Anti-Tyrosinase Activities of Isolated Components

Tyrosinase inhibition assay showed that rosmarinic acid (IC_50_ = 10.12 ± 0.84 μg/mL), quercetin (IC_50_ = 11.20 ± 0.59 μg/mL), *p*-coumaric acid (IC_50_ = 22.20 ± 1.14 μg/mL) and chlorogenic acid (IC_50_ = 79.96 ± 13.00 μg/mL) have good inhibitory effect against tyrosinase, and their IC_50_ values are much lower than that of the positive control, arbutin (IC_50_ = 185 ± 10.56 μg/mL).

### 3.11. Anti-α-Glucosidase Activities of Isolated Components

With further analysis of α-glucosidase inhibitory activity, kaempferol (IC_50_ = 7.88 ± 2.07 μg/mL) and quercetin (IC_50_ = 8.11 ± 1.66 μg/mL) showed strong anti-α-glucosidase activities. In addition, caffeic acid (IC_50_ = 169.69 ± 11.27 μg/mL) and rosmarinic acid (IC_50_ = 185.36 ± 22.76 μg/mL) were more effective than the positive control, acarbose (IC_50_ = 331.24 ± 15.40 μg/mL).

### 3.12. Molecular Modeling Docking Study

According to the experimental data (Table 6), the most potent compounds, rosmarinic acid and kaempferol, were selected to determine their binding abilities to the crystal structures of tyrosinase and α-glucosidase, respectively, by molecular docking.

The 3D crystal structure (PDB: 2Y9X) of tyrosinase from mushroom complexed with tropolone, an inhibitor for tyrosinase, shows that the substrate binding site of tyrosinase is primarily formed by five α-helices as well as several loops, and it mainly contains hydrophilic residues such as six histidines, His 61, 85, 94, 259, 263 and 296, which interact with two copper ions [21]. Once tropolone enters the substrate binding pocket, it is surrounded by some hydrophilic and hydrophobic residues including six histidines, Cys 83, Phe 90, Glu 256, Asn 260, Phe 264, Met 280 and Val 283. Tropolone enters the substrate binding pocket by locating its cycloheptyl core scaffold in the middle of the pocket. It mainly interacts with the pocket by its 1-keto group acting as the H-bond acceptor to interact with His 61 and His 263. Additionally, the cyclohepta-2,4,6-triene scaffold of tropolone makes an essential hydrophobic π-π interaction with His 263. These interactions result in tropolone’s antagonistic activity against tyrosinase.

**Figure 2:**
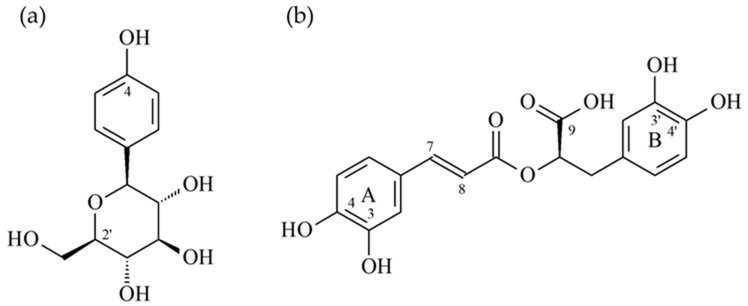
The structures of arbutin (**a**) and rosmarinic acid (**b**).

**Figure 3:**
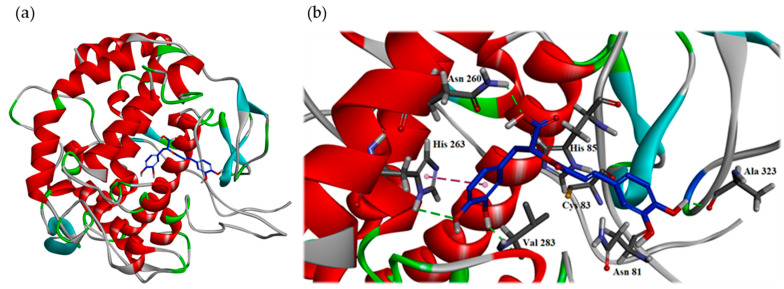
Interaction of rosmarinic acid with the active sites of mushroom tyrosinase. The binding model (**a**) and the hydrophilic interactive model (**b**) of rosmarinic acid in the substrate binding pocket of the crystal structure (PDB: 2Y9X). The carbon atom of rosmarinic acid is in the dark blue color, the carbon atom of protein is in the blue color, the oxygen atom is in the red color and hydrogen atom is in the white color. The green line indicates the hydrogen bond interaction. The pink line indicates the π-π interaction.

To understand how rosmarinic acid (Figure 2b) might make interaction with tyrosinase from mushroom to exert its antagonistic effect, the docking models of rosmarinic acid was generated by the Discovery Studio 2019 (Accelrys, San Diego, CA, USA) CDocker modeling program. The 3D crystal structure (PDB: 2Y9X) of tyrosinase from mushroom was utilized to perform the docking study. In the Figure 3, the docking model of rosmarinic acid showed that rosmarinic acid resided in the substrate binding pocket by locating its 3′,4′-dihydroxyphenylacetic acid moiety to the middle of the pocket as the core scaffold of tropolone, and leaning its A ring toward to the right side of the pocket. By this orientation, rosmarinic acid not only bound to the pocket in the similar manner as tropolone but also exhibited additional interaction with the pocket. As shown in the Figure 3a, the essential interactions rosmarinic acid exerting included (1) 3′-hydroxyl group on the B ring interacted with the backbone of Val 283 by acting as the H-bond donor; (2) the 4′-hydroxyl group on the B ring interacted with His 263 by acting as the H-bond acceptor; (3) the phenyl B ring of rosmarinic acid made the π-π interaction with His 263; (4) the oxygen atom on the 9′-carboxylate group acted as the H-bond acceptor to interact with the side chain of Asn 260; (5) the 4-hydroxyl group on the A ring served as the H-bond donor to contact with the backbone of Ala 323.

As the positive control, the docking model for arbutin (Figure 2a) is also generated to compare its binding mode with rosmarinic acid’s. Arbutin bound to the catalytic site in the similar manner as tropolone by leaning its phenolic ring toward to the tropolone binding position. In the substrate binding pocket, arbutin made three important interactions including (1) the 4-hydroxyl group on the phenolic ring interacted with His 85 by acting as the H-bond donor; (2) the phenolic aromatic ring made the π-π interaction with His 263; (3) the 2′-hydroxymethyl group on the glycosyl ring interacted with Asn 280 by acting as the H-bond acceptor. This model also supported the importance of arbutin’s glycosyl ring due to its contribution to the binding affinity, since deoxyarbutin has been shown to have less binding affinity than arbutin.

The 3′,4′-dihydroxyphenylacetic acid moiety of rosmarinic acid and the phenolic group of arbutin both can locate at the tropolone binding position of tyrosinase’s active site to make the same π-π interaction with His 263 as tropolone. Furthermore, the phenolic group and the glucosyl group of arbutin make two H-bond interactions with His 85 and Asn 260. However, the 3′,4′-dihydroxyphenylacetic acid moiety of rosmarinic acid makes three H-bond interactions with Asn 260, His 263 and Val 283, so rosmarinic acid exerts more hydrophilic interaction than arbutin in the tropolone binding site. Additionally, the caffeic acid moiety of rosmarinic acid makes one H-bond interaction with Ala 323 and can exert weak interaction with the loop of the substrate binding pocket to enhance the binding affinity by its ethenyl group with His 85 and 3-hydroxyl group with Asn 81.

Based on the results mentioned above, it is highly suggested that rosmarinic acid should have better antagonistic activity than arbutin.

The 3D crystal structure of α-glucosidase complexed with acarbose showed that it mainly contains numerous structural domains including N-terminal domain, the barrel domain in which the active site is located and C-terminal domain. The α-glucosidase substrate binding site is primarily formed by numerous β-sheets and several loops or α-helices. The crystal structure of α-glucosidase from *Ruminococcus obeum* complexed with voglibose (PDB: 6C9X) reveals that its active site is formed by six β-sheets, two loops and one α-helice. Once voglibose enters the substrate binding pocket, it is surrounded by some hydrophilic and hydrophobic residues including Pro 75, Trp169, Asp 197, Ile 198, Ile 234, Trp 305, Asp 307, Arg 404, Trp 417, Asp 420, Phe 453 and His 478. Voglibose mainly interacts with the substrate binding pocket by the significant H-bond interactions including when (1) the 1-hydroxymethyl and 2-hydroxyl group interact with Asp 197 by acting as the H-bond donors; (2) the 4-hydroxyl group contacts with Arg 404 by serving as the H-bond acceptor; (3) the 1-hydroxypropan-2yl-amino group makes H-bond interaction with Asp 420.

**Figure 4:**
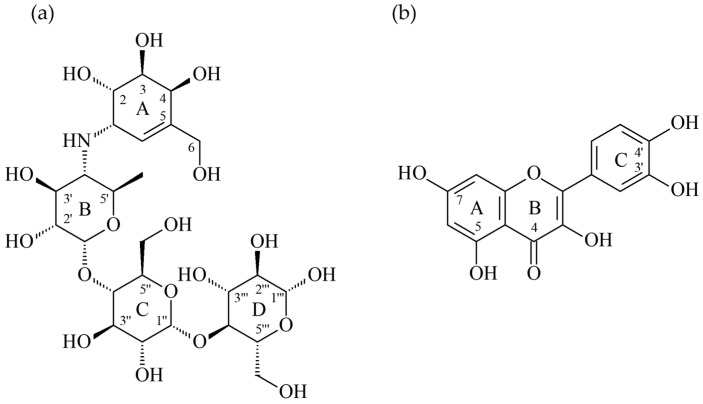
The structures of acarbose (**a**) and kaempferol (**b**).

To further study the interaction between kaempferol (Figure 4b) and the α-glucosidase of *Saccharomyces cerevisiae* and to try to interpret how kaempferol might exert its antagonistic effect, the docking model of kaempferol was generated by the Discovery Studio 2019 (Accelrys, San Diego, USA) CDocker modeling program. The crystal structure of α-glucosidase from *Saccharomyces cerevisiae* is not available now, so the crystal structure (PDB: 3A4A) of *Saccharomyces cerevisiae* containing 72% sequence homology with the α-glucosidase from *Saccharomyces cerevisiae* is usually used to perform the docking study and also employed in this study [22]. In the crystal structure (PDB: 3A4A), the configuration of its substrate binding site is quite similar to that of the α-glucosidase from *Ruminococcus obeum* but it is deep and narrow. In the active site, its co-crystalized ligand, α-D-glucopyranose, locates deeply in the substrate binding pocket and makes three essential H-bond interactions including when (1) the 2-hydroxyl group interacts with Glu 277 and Asp 352 by acting as the H-bond donor; (2) the 3-hydroxyl group makes H-bond contact with Asp 352 by acting as the H-bond donor, and also serves as the H-bond acceptor to interact with Arg 213; (3) the 4-hydroxyl group acts as the H-bond donor to interact with His 351 and Asp 352.

**Figure 5:**
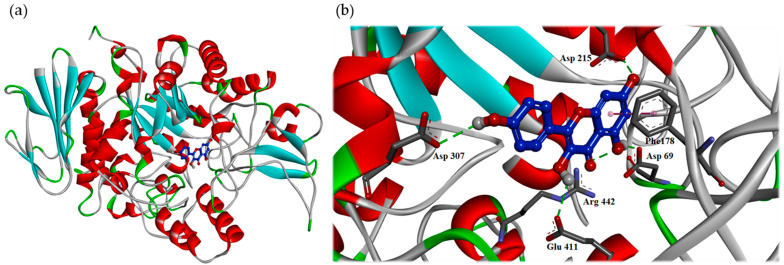
Interaction of kaempferol with active sites of *S. cerevisiae* α-glucosidase. The binding model (**a**) and the hydrophilic interactive mode (**b**) of kaempferol in the substrate binding pocket of the crystal structure (PDB: 3A4A). The carbon atom of kaempferol is in the dark blue color, the carbon atom of protein is in the blue color, the oxygen atom is in the red color and the hydrogen atom is in the white color. The green line indicates the hydrogen bond interaction. The pink line indicates the π-π interaction.

As shown in Figure 5, the docking model of kaempferol indicated that kaempferol did not enter the active site in the flat conformation due to the narrow entrance. Alternatively, the A ring of kaempferol leaned toward to the binding position of α-D-glucopyranose. Importantly, kaempferol made some significant hydrophilic interactions including when (1) the 5-hydroxyl group on the A ring served as the H-bond donor to interact with Asp 69 and acted as the H-bond acceptor to make contact with Arg 442; (2) 7-hydroxyl groups on the A ring behaved as the H-bond donor to contact with Asp 215; (3) the 3-hydroxyl group on the B ring served as the H-bond donor to interact with Glu 411; (4) the 4’-hydroxyl groups on the C ring acted as the H-bond donor to interact with Asp 307. Apart from the hydrophilic interaction, kaempferol also made the important hydrophobic contact, the π-π interaction between its A ring and Phe 178.

As the positive control, the docking model for acarbose (Figure 4a) is also generated to compare its binding mode with kaempferol’s. Acarbose bound to the catalytic site in the similar manner as kaempferol by leaning its A ring toward to the binding position of α-D-glucopyranose and located its D ring to protrude out of the entrance of the active site. Since it contains lots of hydrophilic moieties, acarbose mainly interacted with the active site by the H-bond interactions including when (1) the 3-hydroxymethyl group on the A ring interacted with Asp 215 by acting as the H-bond donor; (2) the 4-hydroxyl group on the A ring interacted with His 351 as well as Asp 352 by acting as the H-bond donor, and also served as the H-bond acceptor to contact with Arg 442; (3) the 6-hydroxymethyl group on the C ring interacted with His 280 by acting as the H-bond donor; (4) the 2-hydroxyl group on the D ring made H-bond interaction with Lys 156 as well as Ser 240 by acting as the H-bond acceptor, and it also made H-bond contact with Leu 313 by acting as the H-bond donor; (5) the 3-hydroxyl group on the D ring interacted with the backbone of Pro 312 as the H-bond donor; (6) the 4-hydroxyl group on the D ring contacted with the side chain of Asp 242 by serving as the H-bond donor.

Unlike acarbose, kaempferol can reside in the middle of the substrate binding pocket and occupy the whole pocket. Especially when kaempferol makes four H-bond interactions with the residues on different secondary structures in the pocket to allow it to stay long enough for exerting its antagonistic effect. For acarbose, its A ring can locate at the similar position as the A ring of kaempferol but its D ring partially protrudes out of the substrate binding pocket. The binding mode of acarbose shows that acarbose highly replies on its A ring to make contact with the residues nearby the substrate binding site. However, the B and C rings of acarbose exert only one hydrophilic interaction. More importantly, the B and C rings of acarbose are surrounded by the hydrophobic residues including Tyr 158, Phe 159 and Phe 178, so their numerous hydroxyl moieties might not be able to exhibit significant hydrophobic interaction as the A ring of kaempferol. Furthermore, the D ring of acarbose protrudes out of the pocket and exerts its interactions with the residues outside the pocket. These interactions out of the substrate binding pocket might not contribute significant binding affinity to the antagonistic effect. All the results mentioned above highly indicate that kaempferol should be able to exert more antagonistic effect than acarbose. However, to generate the crystal structure of the α-glucosidase from *Saccharomyces cerevisiae* complexed with kaempferol or acarbose, further research is needed to further establish the nature of their respective interaction.

**Figure 6:**
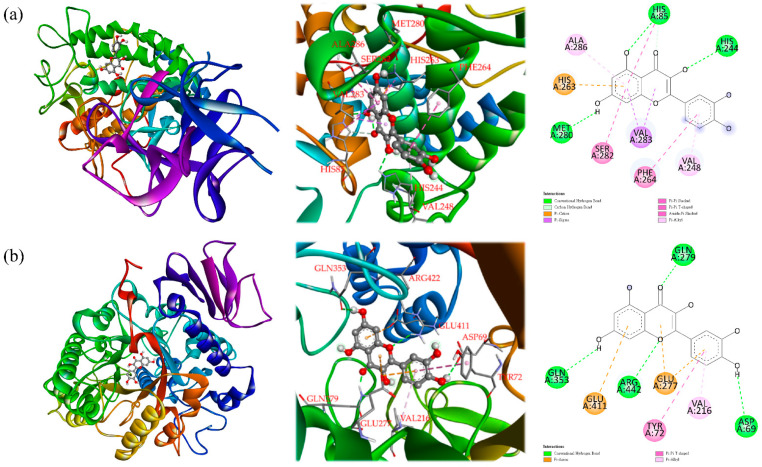
Predicted binding mode of quercetin docked into tyrosinase (**a**) and α-glucosidase (**b**).

In addition, it was found that quercetin exhibited a strong inhibitory effect on both of the tyrosinase and α-glucosidase. As shown in Figure 6, quercetin established hydrogen or carbon hydrogen bonds with HIS 85, HIS 244, and MET 280, and other interactions (π-cation, π-sigma, π-π stacked, π-π T-shaped, amide-π stacked, and π-alkyl) with HIS 263, VAL 283, ALA 286, SER282, PHE 264, and VAL 248 residues of the *Agaricus bisporus* tyrosinase could be detected. Furthermore, quercetin was bound with ASP 69, ARG 442, GLN 279, and GLN353 through conventional hydrogen bonds, while other interactions (π-anion, π-π T-shaped, and π-alkyl) were also observed with GLU 277, GLU411, TYR 72, and Val 216. These allowed quercetin and protein to form a stable complex.

According to the above data, the docking scores of rosmarinic acid, kaempferol, and quercetin were higher than those of arbutin and acarbose, indicating their better binding capability. In this study, the active ingredients, rosmarinic acid, kaempferol and quercetin, possessed not only anti-tyrosinase and anti-α-glucosidase activity, but also the better binding potential with the active sites of *A. bisporus* tyrosinase and *S. cerevisiae* α-glucosidase. This indicated that these compounds may deserve further investigation as natural tyrosinase and α-glucosidase inhibitors.

## 4. Discussion

Several methods have been developed and utilized to extract natural products, i.e., plants, fungi and herbs, as an alternative to modern medicines. The most traditional method applied for many years is to boil or make a decoction with water, which is a very simple and economical procedure. Today, studies based on medicinal plants have been published and many studies are ongoing as many metabolites with health benefits are found in natural products. More recently, in more advanced studies, organic solvents have been used to obtain natural product extracts including various metabolites depending on the polarity and property of the components of interest [23]. Other factors that may affect the natural product extraction process include the type of solvent to be used, the temperature set during extraction, the property of plant material, and the target metabolites [24]. Changes in solvent polarity lead to significant differences in phytochemical composition and biological activity. Therefore, we utilized solvents of various polarities to extract the whole plant of *P. oleracea* in an effort to evaluate these different metabolites. We found that various metabolites have different degrees of biological activity due to differences in solvent polarity.

DPPH and ABTS radical scavenging assays have been widely utilized to assess the antioxidant activities of natural components. Both assays are mainly related to the proton radical scavenging or hydrogen donating ability of the target compound [25]. Superoxide radical scavenging activity is measured by the PMS-NADH-NBT system. Superoxide anion radicals generated from dissolved oxygen by the PMS/NADH coupling reaction reduce NBT. The decrease of absorbance at 560 nm with antioxidants indicates the reduction of superoxide anion radicals in the reaction mixture [26]. The ferric reducing antioxidant power (FRAP) measures the antioxidant potential of each extract through the reduction of ferric iron (Fe^3+^) complex to ferrous iron (Fe^2+^) complex by antioxidants present in the samples [27]. In our study, the ethanolic extract of the whole plant of *P. oleracea* showed high antioxidant activities among all solvent extracts via DPPH and hydroxyl radical scavenging assays. The differences in antioxidant capacities of the extracts may be owing to the different extents of TPC or the composition of antioxidant compounds in the extracts.

The comparative evaluation of the total phenolic content (TPC), total flavonoid content (TFC), and antioxidant assays (DPPH, ABTS, superoxide, and hydroxyl radical) of various solvent extracts (*n*-hexane, chloroform, dichloromethane, EtOAc, acetone, EtOH, and MeOH) from the whole plant of *P. oleracea* is first mentioned in this study. This can provide a guide for the selection of appropriate solvents in TPC, TFC, and antioxidant extraction applications. According to the antioxidant data, rosmarinic acid and quercetin displayed potent antioxidant properties. In addition, the contents of rosmarinic acid and quercetin in the ethanolic extract were the highest among all solvent extracts. This was consistent with the result that the ethanolic extract has high antioxidant activity.

Tyrosinase is an enzyme mainly involved in the biosynthesis of melanin and catalyzes melanin biosynthesis in human skin, which causes diseases of epidermal hyperpigmentation [10,11]. Anti-tyrosinase agent could suppress the activity of tyrosinase. In addition, antioxidants can prevent or delay pigmentation by different mechanisms, e.g., by scavenging ROS and RNS, or by reducing *o*-quinones or other intermediates in melanin biosynthesis, thus delaying oxidative polymerization [28]. Based on our data, rosmarinic acid showed strong antioxidant and anti-tyrosinase activities and was supposed to possess some potential in treatment of diseases related to melanin.

Anti-α-glucosidase agent could suppress the activity of glucosidases in small intestines which split the glycosidic bonds in carbohydrate so that it decreases the glucose release from food. The inhibitors studied were classified into non-sugar and sugar-mimicking types on the basis of their chemical structure. The anti-α-glucosidase drugs for clinical treatment such as voglibose, acarbose, and miglitol all belong to the sugar-mimicking type. Nevertheless, the α-glucosidase inhibitors with the non-sugar type have received the attention of investigators due to the limitations of sugar-mimicking inhibitors. According to the results of an anti-α-glucosidase assay, kaempferol exhibited the most potent anti-α-glucosidase activity among all isolated compounds. Thus, the interaction between α-glucosidase and kaempferol was evaluated by molecular modeling docking. As the result of molecular docking, kaempferol exhibited high affinity with α-glucosidase. Similar experimental results could also be found in past studies [29].

## 5. Conclusions

Various solvent extracts of *P. oleracea* were investigated with various antioxidant systems, anti-tyrosinase and anti-α-glucosidase activity assays. In our study, the ethanol extract of *P. oleracea* displayed the highest total phenol contents (TPC) and relatively high total flavonoid contents (TFC). The overall antioxidant capacity of the ethanolic extract of *P. oleracea* was superior to that of the other solvent extracts, consistent with the results of TPC and TFC assay. Furthermore, the acetone extract of *P. oleracea* showed the highest anti-α-glucosidase activity among all solvent extracts. EtOAc and acetone extracts of *P. oleracea* showed more potent anti-tyrosinase activity than other solvent extracts.

Seven active compounds from *P. oleracea* were quantified by HPLC and identified as chlorogenic acid (**1**), *p*-coumaric acid (**2**), caffeic acid (**3**), *trans*-ferulic acid (**4**), quercetin (**5**), rosmarinic acid (**6**), and kaempferol (**7**). Moreover, the comparative evaluation for the identification and quantification of seven active components of different solvent extracts (*n*-hexane, chloroform, dichloromethane, EtOAc, acetone, EtOH, and MeOH) from the whole plant of *P. oleracea* by HPLC analysis is first conducted in our study.

Biological activity analysis showed that these seven compounds had potent antioxidant activity in general on the scavenging of DPPH, ABTS, superoxide and hydroxyl radicals, especially rosmarinic acid, kaempferol and quercetin. Kaempferol and quercetin showed strong anti-α-glucosidase activity. Rosmarinic acid, quercetin, and *p*-coumaric acid had a potent inhibitory effect against tyrosinase. Further molecular model docking analysis also confirmed their binding sites and binding abilities.

In conclusion, this study demonstrated that extraction solvent for *P. oleracea* affects total phenolic and flavonoid contents, antioxidant activities, and bioactive component levels. Ethanol is the most suitable solvent for extracting the effective components of *P. oleracea* among all solvents. The ethanol extract and its active components (especially rosmarinic acid, kaempferol and quercetin) of *P. oleracea* can be used as natural antioxidant, anti-tyrosinase, and anti-α-glucosidase agents.

## Figures and Tables

**Table 1:** Total phenol contents (TPC), total flavonoid contents (TFC) and extraction yields of *Portulaca oleracea* for each extraction solvent.

Extracting Solvents	Relative Polarity	TPC (mg/g) ^a^ (GAE)	TFC (mg/g) ^b^ (QCE)	Yields (%) ^c^
*n*-Hexane	0.009	19.67 ± 3.33 **	36.41 ± 5.81 **	21.30 ± 2.34
Chloroform	0.259	28.99 ± 1.30 ***	66.60 ± 5.81 **	19.20 ± 1.25
Dichloromethane	0.269	30.12 ± 2.79 **	107.75 ± 7.28 **	20.37 ± 2.11
Ethyl acetate	0.288	30.91 ± 3.09 **	115.49 ± 8.85 **	27.53 ± 2.13
Acetone	0.355	34.61 ± 4.87 **	89.65 ± 8.53 **	31.50 ± 1.56
Methanol	0.762	27.80 ± 2.86 **	27.21 ± 0.74 ***	38.27 ± 1.43
Ethanol	0.654	219.27 ± 4.13 ***	437.38 ± 13.14 ***	33.20 ± 3.65

^a^ TPC was expressed as mg of gallic acid equivalents (GAE) per gram of extract. ^b^ TFC was presented as mg of quercetin equivalents (QCE) per gram of extract; ^c^ Yield was calculated as % yield = (weight of extract/initial weight of dry sample) × 100; Values are presented as means ± SD (*n* = 3); ** *p* < 0.01; *** *p* < 0.001 compared with the control.

**Table 2:** Antioxidant activities of different solvent extracts from *Portulaca oleracea* measured by DPPH, ABTS, superoxide, and hydroxyl radical scavenging assays.

Extracting Solvents	IC_50_ (μg/mL) ^a^			
	DPPH	ABTS	Superoxide	Hydroxyl
*n*-Hexane	>400	296.79 ± 10.16	14.36 ± 2.17 ***	132.94 ± 12.85
Chloroform	152.46 ± 4.71 *	97.91 ± 7.36 *	6.80 ± 1.77 ***	115.95 ± 7.01
Dichloromethane	116.40 ± 4.50 *	79.03 ± 6.52 *	>400	115.66 ± 17.68
Ethyl acetate	64.24 ± 3.03 **	45.40 ± 6.06 **	>400	68.60 ± 5.76 *
Acetone	49.79 ± 3.34	48.41 ± 3.87 **	>400	85.92 ± 5.99
Methanol	55.36 ± 2.08 *	69.85 ± 6.76 *	>400	38.31 ± 0.54 **
Ethanol	41.33 ± 2.89 *	64.69 ± 3.97 *	>400	23.23 ± 0.28 **
BHT ^b^	39.88 ± 1.30 ***	20.34 ± 1.69 ***	N.A. ^c^	65.90 ± 1.23 *

^a^ The IC_50_ value was defined as half-maximal inhibitory concentration of each free-radical scavenging activity, and was expressed as mean ± SD (*n* = 3); ^b^ Butylated hydroxytoluene (BHT) was used as positive control; ^c^ N.A. indicates not available (poor solubility); * *p* < 0.05, ** *p* < 0.01, and *** *p* < 0.001 compared with the control.

**Table 3:** Tyrosinase and α-glucosidase inhibitory activities of different solvent extracts.

Extracting Solvents	IC_50_ (μg/mL) ^a^	
	Tyrosinase	α-Glucosidase
*n*-Hexane	621.43 ± 41.33	418.31 ± 11.95 *
Chloroform	659.87 ± 24.89	527.06 ± 25.64 *
Dichloromethane	545.29 ± 29.29	416.34 ± 13.58 *
Ethyl acetate	396.20 ± 24.73 *	472.92 ± 2.45 *
Acetone	412.22 ± 9.52 *	328.24 ± 52.67 **
Methanol	571.35 ± 25.73	>800
Ethanol	497.24 ± 32.99	440.31 ± 5.94 **
Arbutin ^b^	182.93 ± 12.90 **	-
Acarbose ^b^	-	319.52 ± 24.46 *

^a^ The IC_50_ value was defined as half-maximal inhibitory concentration, and was expressed as mean ± SD (*n* = 3); ^b^ Arbutin and acarbose were used as positive controls; * *p* < 0.05 and ** *p* < 0.01 compared with the control.

**Table 4:** Identification and quantification of the major active components from *Portulaca oleracea* in different solvent extracts.

Extracting Solvents	mg/kg							
	Chlorogenic Acid	*p*-Coumaric Acid	Caffeic Acid	*trans*-Ferulic Acid	Quercetin	Rosmarinic Acid	Kaempferol	Total Amount
Methanol	2.04 ± 0.74	0.92 ±0.62	1.24 ± 1.62	2.64 ± 1.63	3.84 ± 1.62	1.43 ± 0.82	4.86 ± 1.68	16.97 ± 1.25
Ethanol	4.05 ± 1.26	3.25 ± 1.28	2.84 ± 1.06	5.12 ± 2.41	8.72 ± 1.32	2.24 ± 1.62	24.68 ± 1.88	50.90 ± 1.55
Acetone	0.49 ± 0.42	0.42 ± 0.34	2.21 ± 1.21	1.83 ± 1.85	1.64 ± 1.64	0.52 ± 0.32	4.32 ± 1.96	11.43 ± 1.11
Ethyl acetate	0.79 ± 0.46	0.64 ± 0.41	0.83 ± 0.38	1.24 ± 1.34	3.02 ± 1.04	0.66 ± 0.12	3.67 ± 0.88	10.85 ± 0.66
Chloroform	2.94 ± 0.65	1.16 ± 0.48	2.62 ± 1.23	3.63 ± 4.36	1.42 ± 0.32	1.85 ± 0.66	5.22 ± 6.41	18.84 ± 2.02
Dichloro- methane	0.42 ± 0.42	0.84 ± 0.43	2.04 ± 0.63	1.86 ± 1.42	3.08 ± 1.28	0.53 ± 0.48	3.24 ± 0.68	12.01 ± 0.76
*n*-Hexane	1.84 ± 0.48	1.83 ± 0.46	2.54 ± 0.65	3.79 ± 1.36	1.76 ± 0.46	1.45 ± 0.67	3.54 ± 1.28	16.75 ± 0.77

Results are expressed as milligrams of each compound in kilograms of extract.

**Table 5:** Antioxidant activities of isolated components of *Portulaca oleracea* were determined by DPPH, ABTS, superoxide and hydroxyl radical scavenging assays.

Compounds	IC_50_ (μg/mL) ^a^			
	DPPH	ABTS	Superoxide	Hydroxyl
Chlorogenic acid	2.37 ± 0.12 ***	22.57 ± 6.51 *	74.25 ± 8.42 **	205.70 ± 70.55
*p*-Coumaric acid	204.13 ± 9.78 **	27.01 ± 6.12 ***	256.00 ± 16.27 *	60.70 ± 69.24 **
Caffeic acid	0.81 ± 0.09 ***	27.85 ± 18.95 **	57.34 ± 7.44**	153.04 ± 85.84 *
*trans*-Ferulic acid	3.81 ± 0.18 ***	24.47 ± 16.45 ***	541.00 ± 26.95	68.26 ± 65.58 **
Quercetin	1.07 ± 0.13 ***	11.46 ± 1.82 ***	44.08 ± 3.07 ***	9.75 ± 4.39 ***
Rosmarinic acid	1.87 ± 0.09 ***	27.11 ± 14.00 **	61.62 ± 6.43 **	6.56 ± 4.77 ***
Kaempferol	2.32 ± 0.17 ***	8.75 ± 0.90 ***	541.00 ± 18.47	11.46 ± 5.60 ***
BHT ^b^	31.04 ± 2.12 ***	19.34 ± 2.18 **	N.A. ^c^	58.62 ± 2.75 **

^a^ IC_50_ values were defined as the half-maximal inhibitory concentration of each free radical scavenging activity and were expressed as mean ± SD (*n* = 3); ^b^ Butylated hydroxytoluene (BHT) was used as positive control; ^c^ N.A. indicates not available (poor solubility); * *p* < 0.05, ** *p* < 0.01, and *** *p* < 0.001 compared with the control.

**Table 6:** Tyrosinase and α-glucosidase inhibitory activities of different isolated components.

Compounds	IC_50_ (μg/mL) ^a^	
	Tyrosinase	α-Glucosidase
Chlorogenic acid	79.96 ± 13.00 **	>400
*p*-Coumaric acid	22.20 ± 1.14 **	>400
Caffeic acid	>400	169.69 ± 11.27 **
*trans*-Ferulic acid	>400	>400
Quercetin	11.20 ± 0.59 ***	8.11 ± 1.66 ***
Rosmarinic acid	10.12 ± 0.84 ***	185.36 ± 22.76 *
Kaempferol	>400	7.88 ± 2.07 ***
Arbutin ^b^	185 ± 10.56 *	-
Acarbose ^b^	-	331.24 ± 15.40 *

^a^ The IC_50_ value was defined as half-maximal inhibitory concentration, and was expressed as mean ± SD (*n* = 3); ^b^ Arbutin and acarbose were used as positive controls; * *p* < 0.05, ** *p* < 0.01, and *** *p* < 0.001 compared with the control.

## Data Availability

The data presented in this study are available in the main text and the Supplementary Materials of this article.

## Supplementary Materials

The following supporting information can be downloaded at: https://www.mdpi.com/article/10.3390/antiox11020398/s1. Table S1: Retention time, LODs, LOQs, and regression analysis for seven components in *Portulaca oleracea* in reverse phase. Figure S1: Reverse-phase HPLC chromatogram of isolated compounds. Figure S2: Reverse-phase HPLC chromatogram of methanol extract. Figure S3: Reverse-phase HPLC chromatogram of ethanol extract. Figure S4: Reverse-phase HPLC chromatogram of acetone extract. Figure S5: Reverse-phase HPLC chromatogram of ethyl acetate extract. Figure S6: Reverse-phase HPLC chromatogram of dichloromethane extract. Figure S7: Reverse-phase HPLC chromatogram of chloroform extract. Figure S8: Reverse-phase HPLC chromatogram of *n*-hexane extract.
