# Supplementary material for: Comparison of Various Solvent Extracts and Major Bioactive Components from Portulaca oleracea for Antioxidant, Anti-Tyrosinase, and Anti-α-Glucosidase Activities
Source: Antioxidants (Basel). 2022 Feb 16;11(2):398. doi: 10.3390/antiox11020398 (PMC8869629; doi:10.3390/antiox11020398)
Supplement: Supplementary file 1 [file antioxidants-11-00398-s001.zip › antioxidants-1606096-supplementary.pdf]

Supplementary data

Comparison of Various Solvent Extracts and Major Bioactive Components from *Portulaca oleracea* for Antioxidant, Anti-tyrosinase, and Anti-α-glucosidase Activities

Wei-Cheng Chen [1,2,†], Shih-Wei Wang [1,3,4,†], Cai-Wei Li [5], Hsiang-Ru Lin [6], Chang-Syun Yang [7], Yi-Cheng Chu [5], Tzong-Huei Lee [8,*], Jih-Jung Chen [7,9,*]

[1] Department of Medicine, MacKay Medical College, New Taipei City 252, Taiwan; wchena7648@gmail.com (W.-C. C.); shihwei@mmc.edu.tw (S.-W.W.)
[2] Division of Sports Medicine & Surgery, Department of Orthopedic Surgery, MacKay Memorial Hospital, Taipei 104, Taiwan
[3] Institute of Biomedical Sciences, MacKay Medical College, New Taipei City 252, Taiwan
[4] Graduate Institute of Natural Products, Kaohsiung Medical University, Kaohsiung 807, Taiwan
[5] Institute of Traditional Medicine, National Yang Ming Chiao Tung University, Taipei 112, Taiwan; leecw1219.y@nycu.edu.tw (C.-W.L.); chuyc.md07@nycu.edu.tw (Y.-C.C.)
[6] Department of Chemistry, College of Science, National Kaohsiung Normal University, Kaohsiung 824, Taiwan; t3136@nknu.edu.tw
[7] Department of Pharmacy, School of Pharmaceutical Sciences, National Yang Ming Chiao Tung University, Taipei 112, Taiwan; tim0619@nycu.edu.tw (C.-S.Y.)
[8] Institute of Fisheries Science, National Taiwan University, Taipei 106, Taiwan
[9] Department of Medical Research, China Medical University Hospital, China Medical University, Taichung 404, Taiwan
* Correspondence: jjungchen@nycu.edu.tw; Tel.: +886-2-2826-7195; Fax: +886-2-2823-2940
† Authors have contributed equally in this manuscript.

Contents

Table S1. Retention time, LODs, LOQs, and regression analysis for seven components in *Portulaca oleracea* in reverse phase HPLC............S3

Figure S1. Reverse-phase HPLC chromatogram of isolated compounds............S3

Figure S2. Reverse-phase HPLC chromatogram of methanol extract............S4

Figure S3. Reverse-phase HPLC chromatogram of ethanol extract............S4

Figure S4. Reverse-phase HPLC chromatogram of acetone extract............S4

Figure S5. Reverse-phase HPLC chromatogram of ethyl acetate extract............S5

Figure S6. Reverse-phase HPLC chromatogram of dichloromethane extract............S5

Figure S7. Reverse-phase HPLC chromatogram of chloroform extract............S5

Figure S8. Reverse-phase HPLC chromatogram of *n*-hexane extract............S6

Table S1. Retention time, LODs, LOQs, and regression analysis for seven components in *Portulaca oleracea* in reverse-phase HPLC.

Compounds	T_m (min) [a]	Regression equation	Correlation coefficient	LOD (μg/mL) [a]	LOQ (μg/mL) [a]
Rosmarinic acid	21.9	y = 48958x + 3675.1	0.9996	0.19	0.53
Chlorogenic acid	23.8	y = 45096x – 3594.4	0.9999	0.15	0.50
p-Coumaric acid	26.2	y = 83338x + 1645.5	0.9993	0.20	0.63
Caffeic acid	28.0	y = 75513x – 724.3	0.9997	0.15	0.52
trans-Ferulic acid	42.1	y = 130798x – 7125.5	0.9999	0.10	0.48
Quercetin	52.3	y = 27262x + 957.4	0.9998	0.14	0.65
Kaempferol	63.1	y = 29758x + 1079.9	0.9997	0.20	0.65

[a] T_m: Retention time; LOD: Limit of detection; LOQ: Limit of quantification

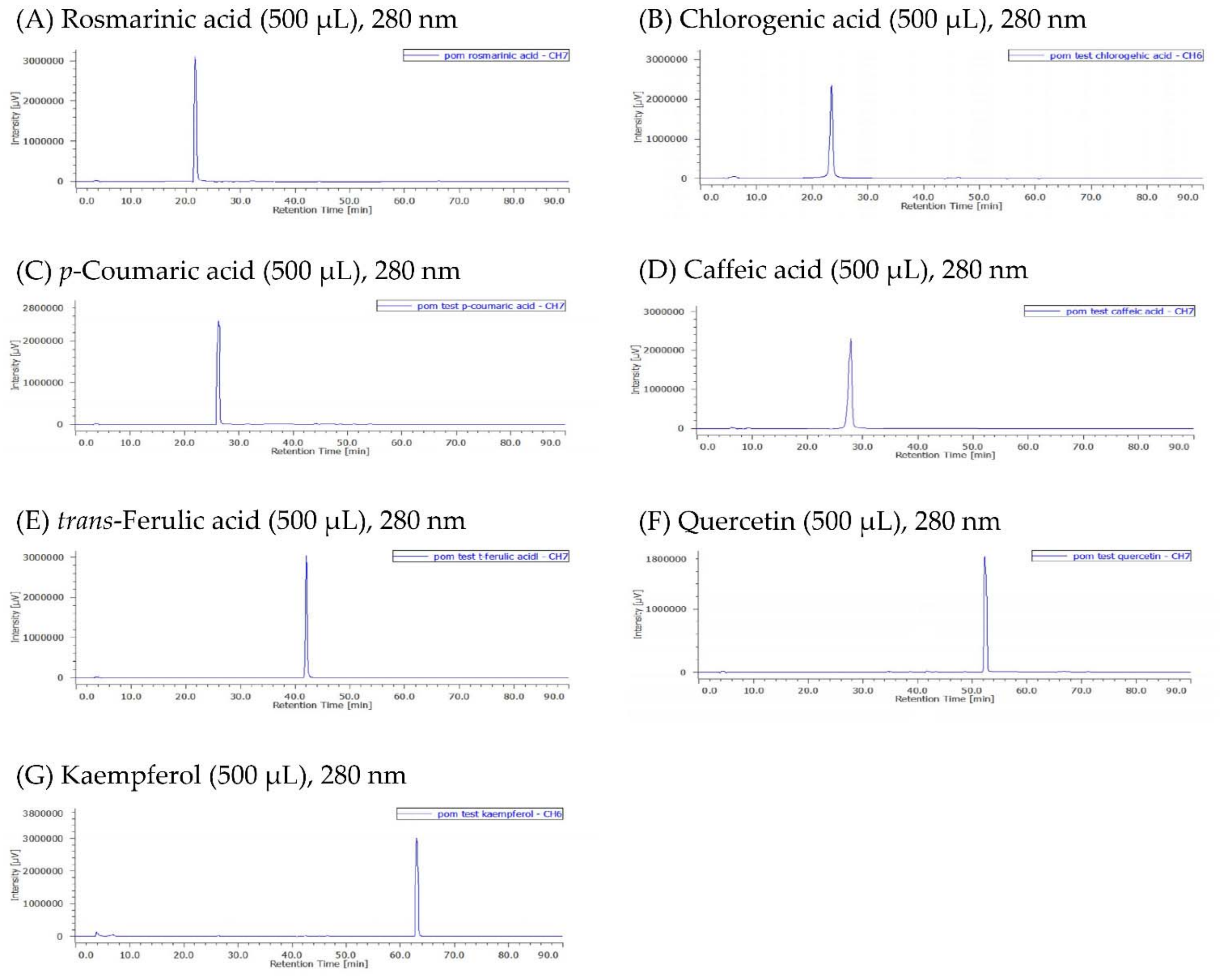

Figure S1. Reverse-phase HPLC chromatogram of isolated compounds (A to G).

Methanol (500 µL), 280 nm

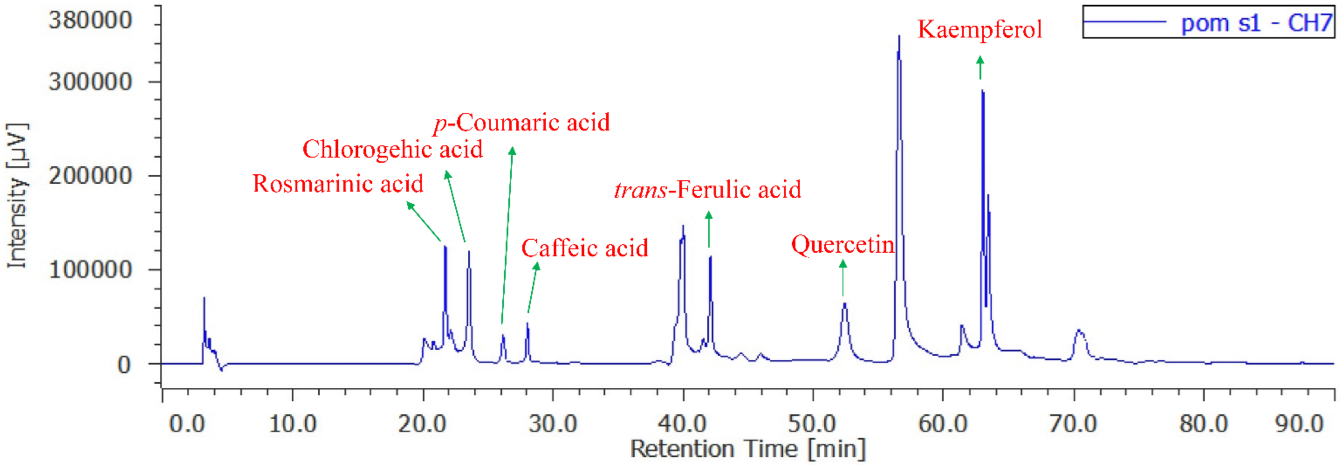

Figure S2. Reverse-phase HPLC chromatogram of methanol extract.

Ethanol (500 µL), 280 nm

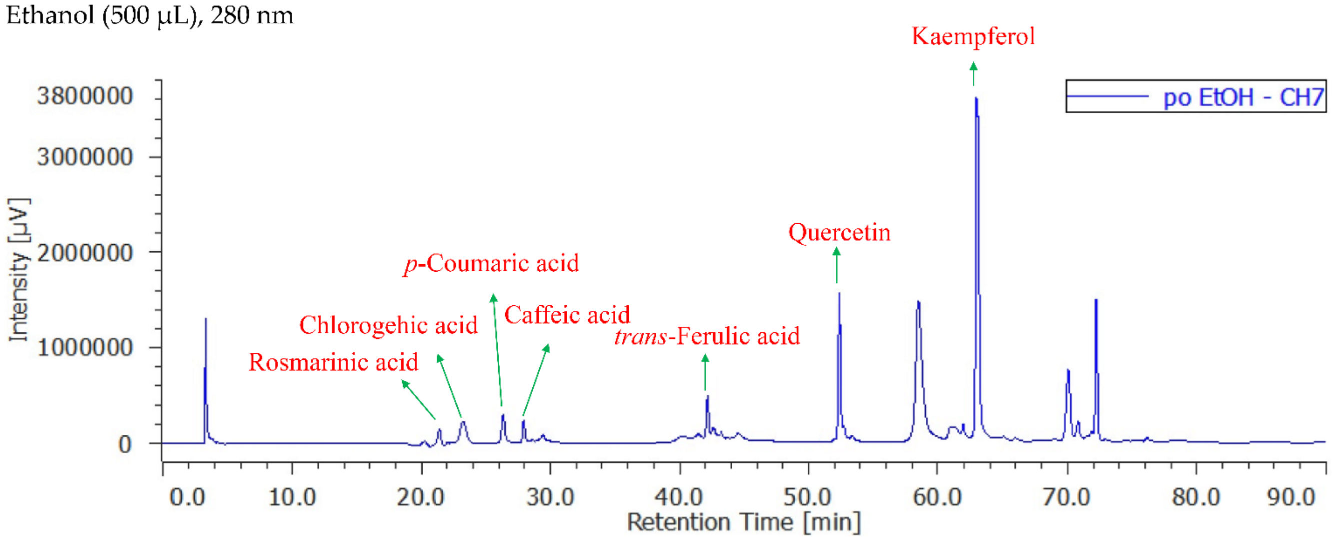

Figure S3. Reverse-phase HPLC chromatogram of ethanol extract.

Acetone (500 µL), 280 nm

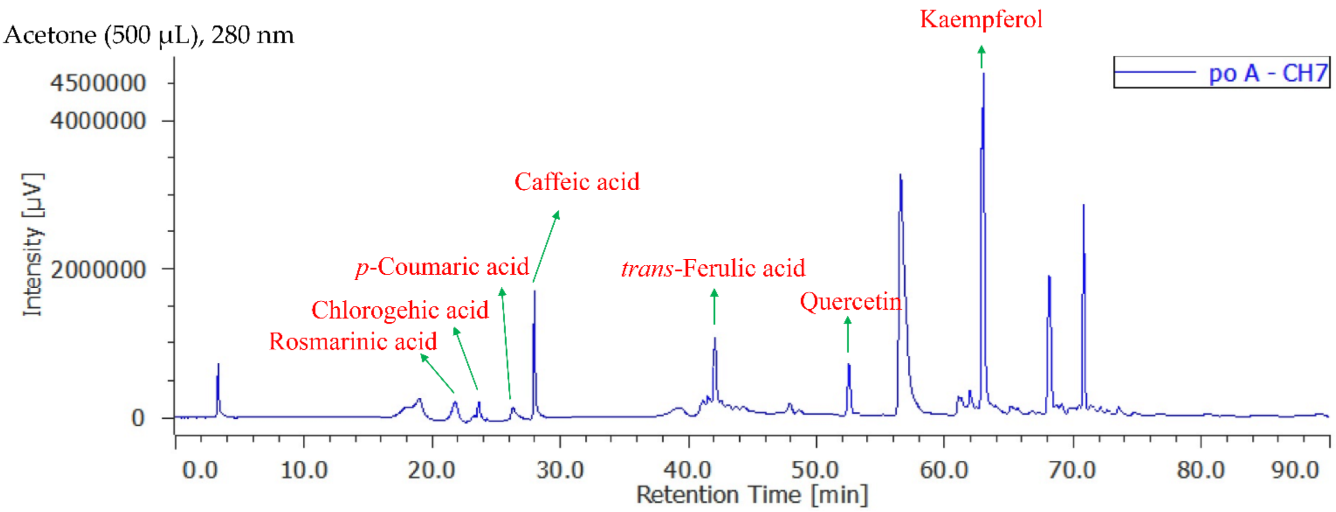

Figure S4. Reverse-phase HPLC chromatogram of acetone extract.

Ethyl acetate (500 μL), 280 nm

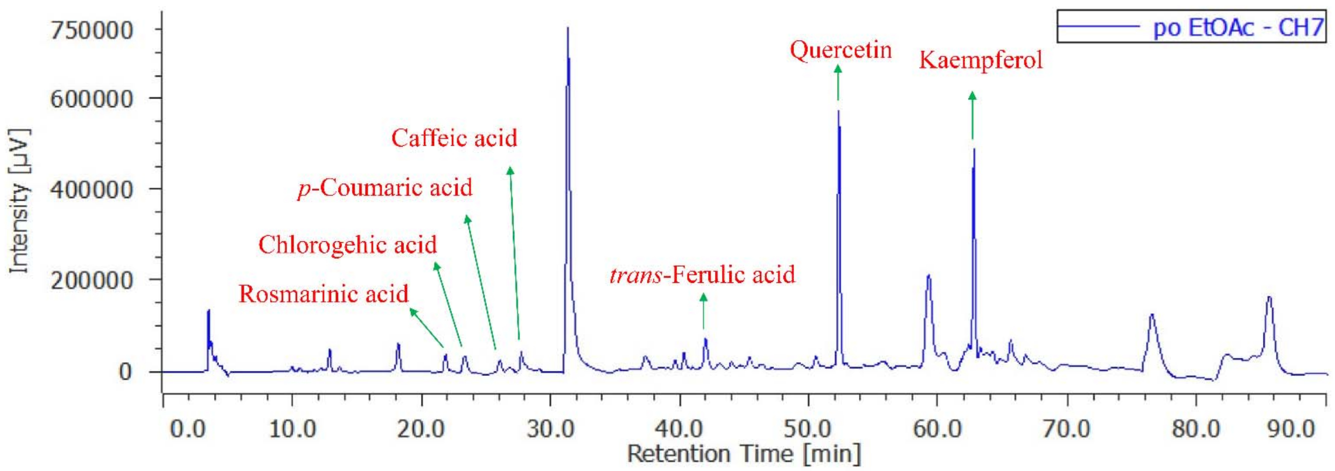

Figure S5. Reverse-phase HPLC chromatogram of ethyl acetate extract.

Chloroform (500 μL), 280 nm

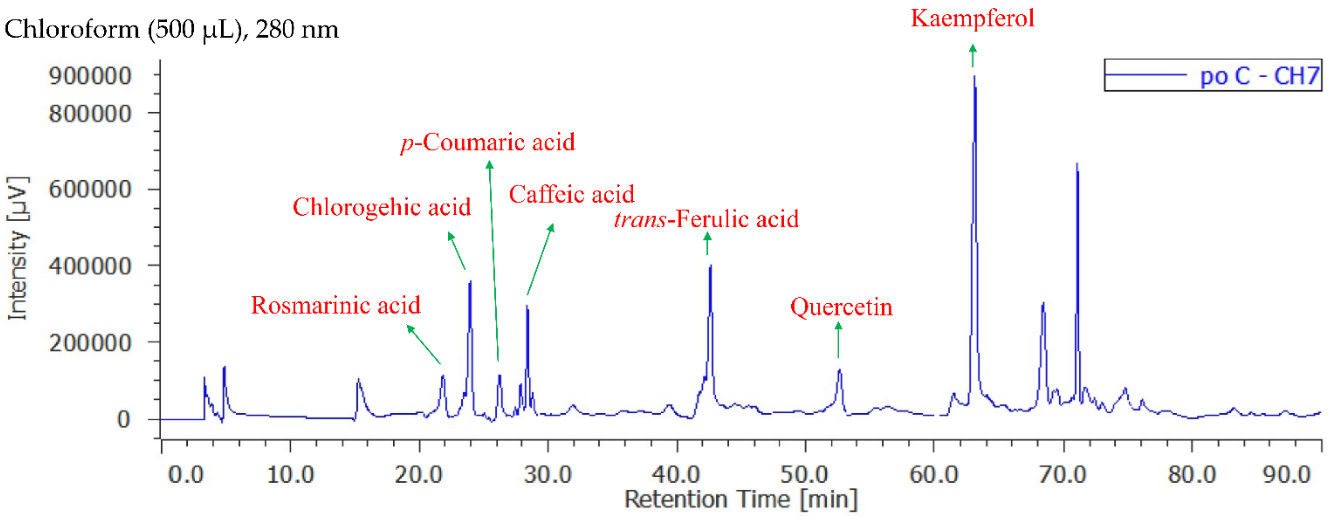

Figure S6. Reverse-phase HPLC chromatogram of dichloromethane extract.

Dichloromethane (500 μL), 280 nm

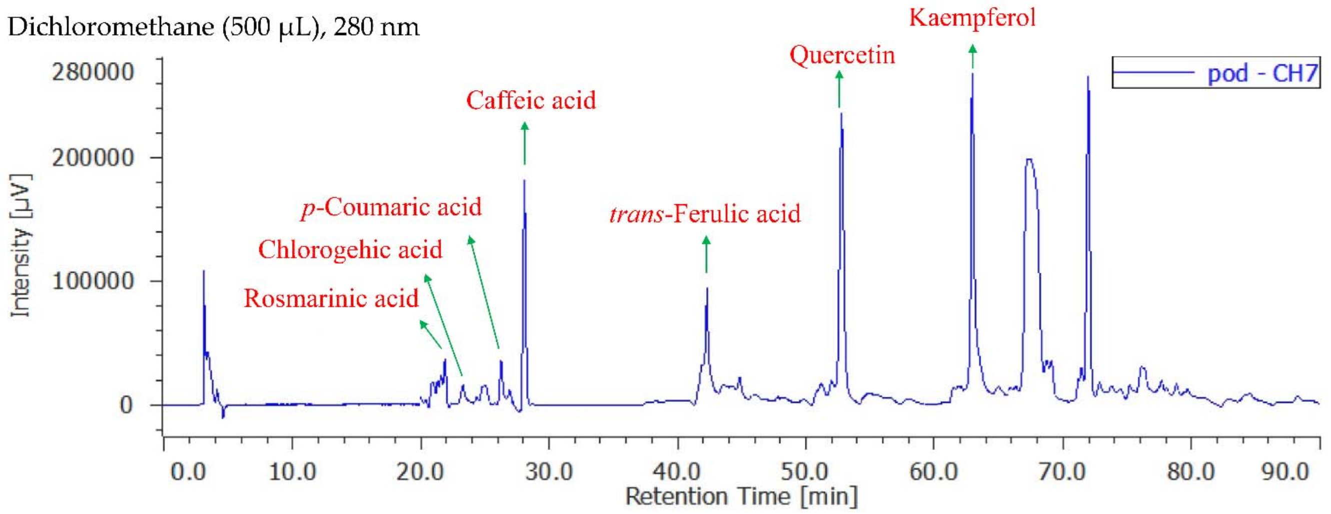

Figure S7. Reverse-phase HPLC chromatogram of chloroform extract.

n-Hexane (500 µL), 280 nm

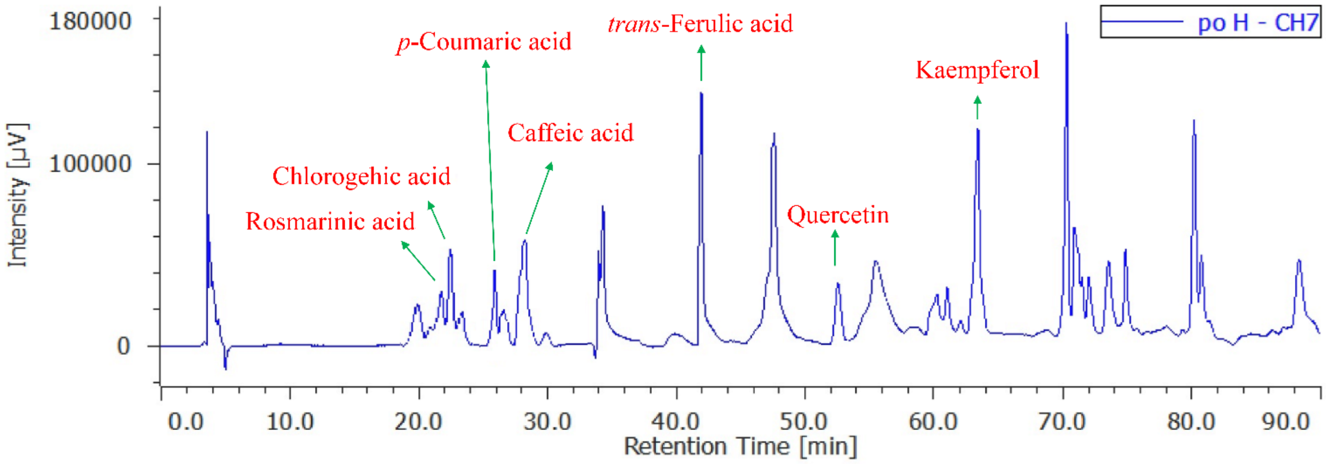

Figure S8. Reverse-phase HPLC chromatogram of *n*-hexane extract.
